# In Situ Reduction of Silver Nanoparticles/Urushiol-Based Polybenzoxazine Composite Coatings with Enhanced Antimicrobial and Antifouling Performances

**DOI:** 10.3390/polym16081167

**Published:** 2024-04-21

**Authors:** Jipeng Chen, Xiaoxiao Zheng, Rongkun Jian, Weibin Bai, Guocai Zheng, Zhipeng Xie, Qi Lin, Fengcai Lin, Yanlian Xu

**Affiliations:** 1 Fujian Engineering and Research Center of New Chinese Lacquer Materials, College of Materials and Chemical Engineering, Minjiang University, Fuzhou 350108, China; jpchen@mju.edu.cn (J.C.); xxzheng@mju.edu.cn (X.Z.); 2231@mju.edu.cn (G.Z.); qlin1990@163.com (Q.L.); 2 State Key Laboratory for Marine Corrosion and Protection, Luoyang Ship Material Research Institute (LSMRI), Xiamen 361100, China; zhipengxie1982@163.com; 3 Fujian Key Laboratory of Polymer Materials, Fujian Provincial Key Laboratory of Advanced Oriented Chemical Engineering, College of Chemistry and Materials, Fujian Normal University, Fuzhou 350007, China; jrkht1987@fjnu.edu.cn (R.J.); bai-wb@fjnu.edu.cn (W.B.)

**Keywords:** urushiol-based benzoxazine, AgNPs, in situ reduction, static antifouling, environmentally friendly

## Abstract

Marine anti-fouling coatings represent an efficient approach to prevent and control the marine biofouling. However, a significant amount of antifouling agent is added to improve the static antifouling performance of the coatings, which leads to an issue whereby static antifouling performance conflicts with eco-friendly traits. Herein, this work reports an in situ reduction synthesis of silver nanoparticles (AgNPs) within polymers to produce composite coatings, aiming to solve the aforementioned issue. Firstly, urushiol-based benzoxazine monomers were synthesized by the Mannich reaction, using an eco-friendly natural product urushiol and *n*-octylamine and paraformaldehyde as the reactants. Additionally, AgNPs were obtained through the employment of free radicals formed by phenolic hydroxyl groups in the urushiol-based benzoxazine monomers, achieved by the in situ reduction of silver nitrate in benzoxazine. Then, the urushiol-based benzoxazine/AgNPs composite coatings were prepared by the thermosetting method. AgNPs exhibit broad-spectrum and highly efficient antimicrobial properties, with a low risk to human health and a minimal environmental impact. The composite coating containing a small amount of AgNPs (≤1 wt%) exhibits effective inhibition against various types of bacteria and marine microalgae in static immersion, thereby displaying outstanding antifouling properties. This organic polymer and inorganic nanoparticle composite marine antifouling coating, with its simple preparation method and eco-friendliness, presents an effective solution to the conflict between static antifouling effectiveness and environmental sustainability in marine antifouling coatings.

## 1. Introduction

No man-made surface submerged in water is immune to marine biofouling, which poses a substantial challenge to the development of the global marine industry, maritime transportation, and aquaculture industry, resulting in numerous significant problems and considerable economic losses annually [1,2,3]. Numerous technologies have been employed to tackle the issue of biofouling, yet due to the intricacy of the maritime environment, the variety of fouling organisms and the complexity of the biofouling process, antifouling solutions for marine vessels remain a persistent, worldwide challenge [4,5]. Marine antifouling coatings represent a cost-effective and efficient solution, as they are the most widely utilized method for preventing fouling [6].

In the 1960s, the tributyltin selfpolishing antifouling coating (TBT-SPC) was the most widespread and successful antifouling coating due to the effective, broad-spectrum and long-lasting antifouling property [7]. However, TBT-SPC has been globally banned because of its high toxicity to other marine organisms and the adverse impact of toxic substance accumulation in the biological chain, which has caused serious damage to the marine ecosystems and environment [8,9]. Currently, copper-based antifouling coatings with cuprous oxide (Cu_2_O) as the primary antifouling agent are the most effective commercially available options, in which the content of Cu_2_O was higher than 40% [10]. Cu_2_O releases slowly from the coatings and experiences a disproportionation reaction that produces Cu^2+^ ions, which eliminate fouling organisms in the marine environment [10]. This is also an effective and simple antifouling strategy, but it has been found that large amounts of Cu^2+^ released into the marine environment can cause many environmental problems [11]. For instance, Cu^2+^ can accumulate in the marine environment or in organisms, affecting non-target organisms, which can also accumulate through the food chain, thereby affecting the human living environment. The widespread use of Cu_2_O has led to the development of copper-resistance in certain fouling organisms, which in turn reduces the efficacy of antifouling coatings [12]. As a result, auxiliary antifouling agents containing toxic and harmful organic booster biocides are often added to Cu_2_O-based antifouling coatings in order to improve its antifouling performance [12]. However, this practice poses the same environmental risks as Cu_2_O and, in some cases, may even present greater hazards. As science and environmental awareness progress, the toxic anti-fouling coatings will be gradually eliminated due to their damaging impact on the marine ecosystem, which will concurrently foster the advancement of non-toxic or low-toxic, eco-friendly alternatives.

Silver nanoparticles (AgNPs) possess highly effective and broad-spectrum antimicrobial characteristics and are extensively applied in the fields of water treatment [13,14], biomedicine [15,16], and food preservation [17,18]. AgNPs have potent antibacterial properties against both Gram-negative and Gram-positive bacteria, as well as various drug-resistant bacteria, at low concentrations due to their minute size and ability to permeate cell membranes [4]. The antimicrobial properties of AgNPs are a result of their direct interaction with bacteria and the indirect interaction mediated by Ag^+^ ions released from their surfaces [4,19]. AgNPs and Ag^+^ can both reach the cell wall of bacteria, causing disruption of the cell membrane and leading to a loss of cell membrane integrity and cell lysis [20]. In view of the environmentally friendly and non-hazardous nature [21,22], AgNPs offer a wide range of potential applications as antifouling agents, which align with green and eco-friendly practices for antifouling. However, as nanomaterials have an inherent interfacial problem of easy agglomeration, surface modification is necessary.

The principal constituents of marine antifouling coatings are antifouling agents and antifouling resins. The development of high-performance and eco-friendly antifouling resins is another current focal point in marine antifouling research [23]. Notable prominent marine antifouling resins that exhibit outstanding performance and are eco-friendly features encompass degradable polymer antifouling resins [24,25], biomimetic materials [26,27], hydrophilic protein-resistant polymers [28,29] and low-surface-energy marine antifouling materials [30,31]. However, various antifouling resins encounter problems including a poor static antifouling effect [32]. Antifouling agents or antifouling groups are an efficient solution for addressing the poor static antifouling of resins [33]. The urushiol-based benzoxazine polymer, as a marine antifouling coating reported by our team, has low surface energy; nevertheless, there is the issue of an insufficient static antifouling effect [34,35]. The raw lacquer-derived renewable natural product, urushiol, includes a special catechol structure that can initiate a semiquinone radical with high activity, which can reduce silver ions to silver monomers [36].

In consideration of the reductive nature of semiquinone radicals, an urushiol-based benzoxazine polymer/nanosilver composite coating was prepared by the in situ reduction of urushiol found in urushiol-based benzoxazine monomers. By coordinating with O and N elements in benzoxazine, AgNPs were able to be uniformly dispersed in polymers through the in situ reduction process, effectively overcoming the tendency of nanoparticles to agglomerate. In the composite coating, the benzoxazine polymer serves multifunctional roles as a reducing agent, dispersant and stabilizer. AgNPs, an eco-friendly antifouling agent, fulfil the needs of eco-friendly marine antifouling coatings and successfully address the issue of an inadequate static antifouling effect of antifouling coatings. Furthermore, the antifouling performance of the urushiol-based benzoxazine polymer/nanosilver composite coatings was tested against prevalent fouling agents including bacteria (*Escherichia coli*, *Staphylococcus aureus*, and marine bacteria *Vibrio alginolyticus* and *Bacillus* sp.) and marine microalgae (*Nitzschia closterium*, *Phaeodactylum tricornutum* and *Dicrateria zhanjiangensis*). This work presents a simple in situ reduction method for preparing urushiol-based benzoxazine polymer/nanosilver composite coatings. UOB serves multiple functions in this work, acting as a reducing agent for AgNP preparation, a dispersant to prevent agglomeration of AgNPs, and a film-forming substrate to provide a low-surface-energy coating with fouling release properties. In addition, AgNPs act as an antifouling agent to effectively kill (repel) fouling organisms. Therefore, the composite coating has multiple functions and exhibits good synergistic antifouling performance. The composite coatings made from organic polymers and inorganic nanoparticles in this study offer the benefits of simple operation and excellent nanoparticle dispersion, which provide both practical and theoretical support for overcoming the issue of insufficient static antifouling effectiveness in marine coatings.

## 2. Materials and Methods

### 2.1. Materials

**Figure 1:**
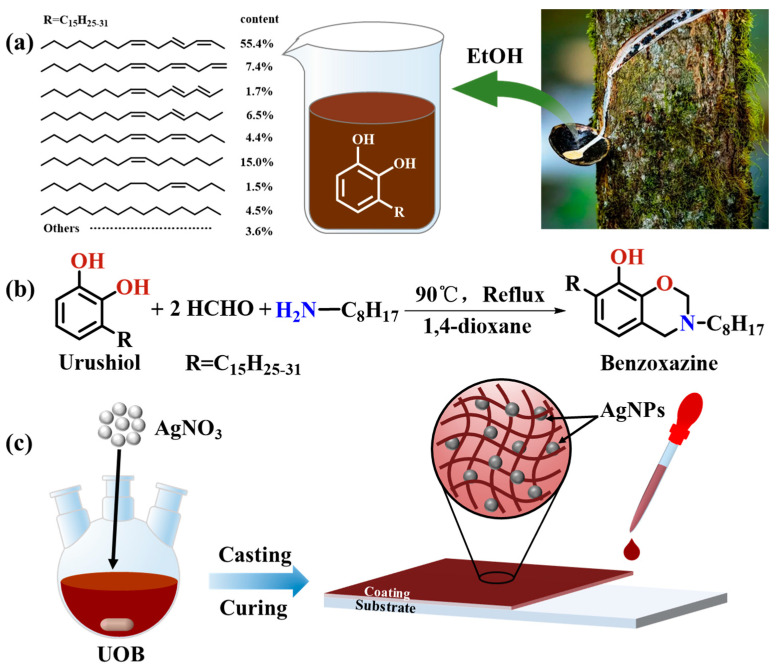
(**a**) Collection of raw lacquer and chemical structure of urushiol, (**b**) synthetic route of urushiol-based benzoxazine, (**c**) preparation of UOB/AgNPs composites and UOHP/AgNPs composite coatings.

Unless otherwise specified, all compounds were used without further purification. The Chinese lacquer used in this study was purchased from Maoba Town, Hubei Province, China. As described in the literature [37], urushiol were extracted from Chinese lacquer using ethanol, and the chemical structure of urushiol is shown in Figure 1a. Analytical grade ethanol, anhydrous sodium sulfate, 1,4-dioxane, dichloromethane and xylene were purchased from Sinopharm Chemical Reagent Co., Ltd. (Shanghai, China). Paraformaldehyde (analytical grade) and *n*-octylamine (99%) were purchased from Shanghai Macklin Biochemical Co., Ltd. (Shanghai, China) and Shanghai Aladdin Biochemical Technology Co., Ltd. (Shanghai, China), respectively. Silver nitrate (analytical grade) was purchased from Xilong Scientific Co., Ltd. (Shantou, China). Phosphate-buffered saline (PBS) with a pH of 7.4 was purchased by Shanghai Sangon Biotech Co., Ltd. (Shanghai, China). Deionized (DI) water was purified using a Direct-Q^®^ 3UV lab ultrapure water system (Merck Millipore, Molsheim, France) and was used throughout the work. Artificial seawater (ASW) was prepared following the guidelines of ASTM D1141-1998 (2013) [38]. *Escherichia coli* (*E. coli*), *Staphylococcus aureus* (*S. aureus*) and *Vibrio alginolyticus* (*V. alginolyticus*) were purchased from BeNa Chuanglian Biotechnology Co., Ltd. (Xinyang, China). *Bacillus* sp. was purchased from Marine Culture Collection of China (Xiamen, China). *Nitzschiaclosterium* (*N. closterium*), *Phaeodactylumtricornutum* (*P. tricornutum*) and *Dicrateriazhanjiangensis* (*D. zhan-jiangensis*) were purchased from Seaweed Culture Collection Center, Institute of Oceanology, Chinese Academy of Sciences (Qingdao, China).

### 2.2. Synthesis of Urushiol-Based Benzoxazine Monomers (UB)

Urushiol/*n*-octylamine benzoxazine monomers (UOB) were synthesized according to the previous literature [34,37]. The synthetic route of benzoxazine was shown in Figure 1b. Briefly, urushiol, paraformaldehyde and *n*-octylamine with the mole ratio of 1:2:1 were dissolved in 1,4-dioxane and loaded in a three-necked round bottom flask. The system was then maintained at 90 °C for 5 h under vigorous stirring. Afterwards, the system was successively extracted with dichloromethane, washed with DI water and dried under vacuum at room temperature. The reddish-brown viscous product was obtained and named UOB.

### 2.3. Preparation of Silver Nanoparticles by In Situ Reduction of Urushiol-Based Benzoxazines

Urushiol-based benzoxazine monomers/silver nanoparticle composites (UOB/AgNPs) were prepared by in situ reduction with the following procedure. A certain amount of benzoxazine monomer and xylene were loaded in a beaker and stirred until a homogeneous solution (40 wt%) was obtained. Various masses of silver nitrate were dissolved in methanol under ultrasonication. The xylene solution of benzoxazine underwent a reaction for 2 h at room temperature while being shielded from light, after the gradual addition of the methanol solution of silver nitrate. The UOB/AgNP composites were obtained and named UOB-x%AgNPs according to the content of AgNPs. The specific compositions can be seen in Table 1.

### 2.4. Fabrication of Silver Nanoparticles by In Situ Reduction of Urushiol-Based Benzoxazines Polymer Coatings

Urushiol-based benzoxazine polymers/silver nanoparticle composite coatings (UOHP/AgNPs) were prepared by casting UOB/AgNP composites onto glass slides for surface wettability, antimicrobial testing and algae inhibition testing. Bare glass slides (BG, 2.5 cm × 2.5 cm) were first cleaned with acetone, ethanol and deionized water for 10 min using ultrasonication and then dried under flowing N_2_ to remove any impurities. The UOB/AgNPs composites were cured at room temperature for 1 h to allow the solvent to evaporate. Subsequently, the composites were cured in a convection oven in steps of 100 °C for 1 h, 120 °C for 1 h, 140 °C for 1 h and 160 °C for 1 h. Once the curing process was complete, the samples were allowed to cool slowly to room temperature, resulting in dark-brown films. The composite coatings had an average thickness of approximately 60 μm, as determined by a Qnix^®^ 4500 coating thickness gauge (Qnix, Germany), following ASTM B499-2009 (2014) [39]. The corresponding composite coatings were labelled as UOHP-x%AgNPs according to the composition list and nomenclature rules provided in Table 1.

### 2.5. Characterizations

Silver nanoparticles (AgNPs) synthesized by an in situ reduction in urushiol-based benzoxazine and the chemical structure of the composite were confirmed by UV-visible spectroscopy (UV-vis), attenuated total reflectance Fourier transform infrared (ATR-FTIR), X-ray diffraction (XRD), X-Ray photoelectron spectroscopy (XPS), energy dispersive Spectroscopy (EDS) and transmission electron microscopy (TEM). UV-vis absorption spectra for AgNPs were obtained on a TU-1901 UV–visible spectrophotometer (Persee, Beijing, China) following the dilution of the UOB/AgNP composites with anhydrous ethanol. ATR-FTIR spectra of UOB and the composite were recorded on a Nicolet 5700 FTIR spectrometer (Thermo Fisher Scientific, Waltham, MA, USA) in the range of 400–4000 cm^−1^. XRD measurements of UOHP and UOHP/AgNPs composite coatings were conducted on a X’Pert PXRD (Panalytical, The Netherlands) using a Cu Kα target with X-ray radiation at a wavelength of 1.54 Å. The range of testing was from 10° to 90° with a scanning step of 0.02°. XPS was performed on an Escalab Xi+ XPS spectrometer (Thermo Fisher Scientific, Waltham, MA, USA). The elemental composition and electron binding energy of UOHP and UOHP/AgNPs composite coating surfaces were recorded, and all the binding energies were calibrated by C 1s peak at 284.6 eV. EDS was measured on a scanning electron microscope (SU8010, Hitachi, Tokyo, Japan) equipped with an Inca250 energy dispersive X-ray analyzer (Oxford, UK) at an accelerating voltage of 15 kV. The morphology of AgNPs in UOHP-1.0%AgNP composite coatings was observed by TEM (FEI Talos 200s, Thermo Fisher Scientific, Waltham, MA, USA). The UOHP-1.0%AgNP composite coatings were sliced using an EM UC7 ultrathin slicer (Leica, Düsseldorf, Germany) and subsequently underwent high-resolution TEM testing as well as EDS analysis at 200 kV. The thermal stability of the UOHP/AgNP composite coatings was determined using a METTLER TGA2 thermal analyzer (Mettler-Toledo, Greifensee, Switzerland). The analysis was conducted in a nitrogen atmosphere with a heating rate of 10 °C/min over a temperature range of 30 to 600 °C. The water contact angle (WCA) and surface free energy (SFE) of the UOHP/AgNP composite coatings were measured using a droplet shape analyzer DSA 25 instrument (Kruss, Hamburg, Germany) with 2 μL of DI water droplets at ambient temperature. The SFE values were calculated using the EOS model. The average values of WCA and SFE for each sample were calculated from the average of five data.

### 2.6. Antibacterial Performance Assessments

According to the previous procedure [40], the antibacterial performance of the UOHP/AgNPs composite coatings was evaluated using Gram-negative *E. coli* BW25113 and Gram-positive *S. aureus* ATCC 25923 and the marine bacterial species *V. alginolyticus* ATCC 33787 (Gram-negative bacteria) and *Bacillus* sp. MCCC 1B00342 (Gram-positive bacteria). The *E. coli* and *S. aureus* bacterial strains were preserved in a 1:1 solution of Luria-Bertani (LB) broth, 40% (*v*/*v*) glycerol, and stored at −80 °C. Before the antibacterial test, the bacterial strains were incubated in a fresh LB broth at 37 °C with shaking at 170 rpm until the O.D._600nm_ reached 1.8–2.0 for 20 h. Similarly, the *V. alginolyticus* and *Bacillus* sp. were stored in solution of 2216E medium and glycerol and frozen at −80 °C. Prior to use, *V. alginolyticus* was pre-activated in fresh 2216E medium at 30 °C, while *Bacillus* sp. pre-activated at 28 °C. BG and UOHP/AgNP composite coatings (7.5 cm × 2.5 cm) were wiped with anhydrous ethyl alcohol, sterilized with UV radiation for 30 min, and then placed in plastic Petri dishes. The bacterial suspension was diluted with fresh LB broth to a concentration of approximately 10^5^–10^6^ CFU/mL. Subsequently, 500 μL of the diluted bacterial suspension was inoculated onto the BG and UOHP/AgNP composite coatings and covered with plastic film. The bacterial strains were incubated for 24 h under static conditions at different temperatures: 37 °C for *E. coli* and *S. aureus*, 30 °C for *V. alginolyticus* and 28 °C for *Bacillus* sp. After that, the plastic films were removed, and the plates were rinsed with 20 mL of sterile PBS to wash away any un-adhered bacteria. The washed-down bacteria were cultured on agar plates, and the number of colonies was counted. To determine the surface-attached bacteria, the washed BG and UOHP/AgNP composite coatings were examined using FE-SEM (SU8010, Hitachi, Tokyo, Japan).

### 2.7. Algal Inhibition Assessments

Algal attachment experiments were performed to assess the impact of UOHP/AgNP composite coatings on algae including *N. closterium*, *P. tricornutum* and *D. zhan-jiangensis* cells. The algal cells were grown in f/2 medium prepared in ASW at a temperature of 22 ± 2 °C, with a cycle of 12 h of fluorescent light and 12 h of darkness. After 7 days of growth, the algal cells were diluted with fresh f/2 medium to obtain a test medium with an algal cell concentration of 10^5^–10^6^ cells/mL for the following algal attachment experiments. The sterilized BG and UOHP/AgNPs composite coatings, similar to the antibacterial performance assessments, were immersed in 30 mL of medium inoculated with *N. closterium*, *P. tricornutum* and *D. zhan-jiangensis* cells. After 5 days of immersion, the BG and UOHP/AgNPs composite coatings were removed from the algal medium and rinsed with 20 mL of sterile PBS to wash away any un-adhered algae. The algae adhering to the surface of the BG and UOHP/AgNPs composite coatings were then detected using a fluorescence microscope (Eclipse Ci-L plus, Nikon, Tokyo, Japan) equipped with a DAPI fluorescence filter kit. Images of five randomly selected fields (20× magnification, 0.156 mm^2^/per field) were recorded for each sample. The coverage of algal cells on the BG and UOHP/AgNP composite coatings was determined by analyzing fluorescence microscopy images using ImageJ 1.52a software.

## 3. Results and Discussion

### 3.1. Synthesis and Characterization of UOB/AgNP Composites

**Figure 2:**
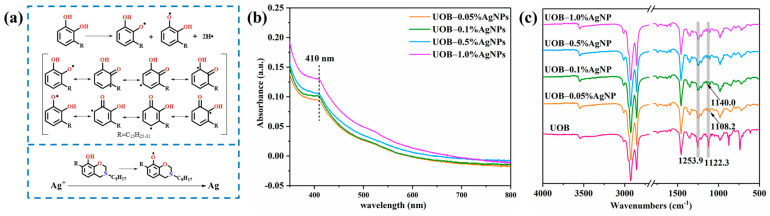
(**a**) Mechanism of free radical formulation in the structure of urushiol and preparation of AgNPs by in situ reduction, (**b**) UV-vis spectra of UOB solution and UOB/AgNP composites, (**c**) ATR-FTIR spectra of UOB and UOB/AgNP composites.

The structure of urushiol-based benzoxazine consists of a catechol structure with a length side chain derived from urushiol. The side chain and the phenolic hydroxyl group on the benzene ring act as electron-donating groups, activating the benzene ring and thus rendering it susceptible to electrophilic reactions. Upon exposure to oxygen, one electron from the phenolic hydroxyl group in urushiol is lost, causing it to transform into a semiquinone radical. Eventually, this radical disproportionates into urushiol and urushiol quinone, as illustrated in Figure 2a. It has been reported in the literature that urushiol and urushiol-based polymers contain highly active free radicals that lead to the reduction of silver ions to obtain silver monomers [36,41].

The UOB solution and UOB/AgNP composites following in situ reduction were evaluated for UV-vis absorption spectra to confirm the presence of AgNPs, and the results are shown in Figure 2b. Based on the previous literature, the absorption peaks on the UV-vis absorption spectra of AgNPs were located at 390~430 nm [42,43,44]. As shown in Figure 2b, the UOB-0.05%AgNPs, UOB-0.1%AgNPs, UOB-0.5%AgNPs and UOB-1.0%AgNP composites all exhibited an absorption peak centered at ca. 410 nm, indicating that the small-sized spherical-like-shaped silver nanoparticles formed [45,46]. In addition, the absorbance varied among the UOB/AgNP composites, indicating a progressive rise with higher AgNPs content. Evidently, there was a gradual increase in the amount of AgNPs produced by in situ reduction within the composites. Therefore, the UV-vis absorption spectra provided preliminary confirmation of AgNP generation through in situ reduction of UOB.

Subsequently, to characterize the structural changes of the UOB and UOB/AgNP composite following in situ reduction, ATR-FTIR spectra of the composite was performed, as shown in Figure 2c. The ATR-FTIR spectra revealed no significant difference in the main characteristic absorption bands of the UOB and UOB/AgNP composite. The ATR-FTIR spectra of the UOB and UOB/AgNP composite featured a broad peak at 3300–3400 cm^−1^ representing free phenolic –OH and characteristic absorption bands centered at 3012.3, 2925.4 and 2853.6 cm^−1^ representing =C–H and C–H stretching vibrations. In addition, the absorption peak at 1745.2 cm^−1^ is assigned to C=O stretching vibrations and the absorption peaks at 1586.6 cm^−1^ and 1456.2 cm^−1^ are assigned to C=C stretching vibrations. Moreover, the characteristic oxazine ring-related band occurred at 964.6 cm^−1^ [34,35]. However, the absorption bands attributed to Ar–O at 1253.9 cm^−1^ and C–N–C at 1122.3 cm^−1^ for UOB differed from those of the UOB/AgNPs composite. In the UOB/AgNP composite, the absorption band of Ar–O changes from a sharp peak to a broad peak, and the absorption band of C–N–C undergoes a significant shift and becomes two small peaks. The ATR-FTIR absorption bands of the UOB/AgNPs composite differ from those of UOB, possibly due to the generation of AgNPs through in situ reduction and their interaction with N and O atoms [36,47,48]. The results further confirm the successful preparation of the UOB/AgNP composite.

### 3.2. Characterization of UOHP/AgNP Composite Coatings

**Figure 3:**
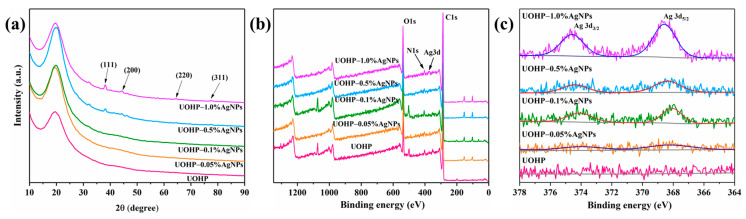
(**a**) XRD patterns of UOHP coating and UOHP/AgNP composite coatings, (**b**) XPS survey of UOHP coating and UOHP/AgNP composite coatings, (**c**) XPS high resolution Ag 3d spectra of UOHP/AgNP composite coatings.

To further confirm the production of AgNPs through the in situ reduction of the UOB monomer, and therefore the presence of AgNPs in the UOHP/AgNP composite coatings, the composite coatings were first characterized by XRD, and the results are shown in Figure 3a. UOHP is a polymer coating lacking a crystalline structure, hence its XRD pattern displays merely one wide packet peak without any additional peaks. The XRD patterns for both UOHP-0.05%AgNP and UOHP-0.1%AgNP composite coatings are not significantly distinct from that of UOHP. This could be attributed to the low content of AgNPs in the composite coatings, subsequently leading to no discernible peaks in the XRD patterns. However, new peaks were clearly observed in the XRD patterns of UOHP-0.5%AgNP and UOHP-1.0%AgNP composite coatings, with peak intensity increasing proportionately as the AgNP content increased. These results suggest that new crystal structures appeared in the composite coatings. Specifically, four peaks at the 2θ positions of 38.2°, 44.3°, 64.5° and 77.5° were assigned to diffraction from the (111), (200), (220) and (311) planes of face cubic crystal AgNPs [45,46,49]. The XRD results provided preliminary evidence of AgNPs presence in UOHP/AgNP composite coatings. It signifies the reduction of AgNPs in situ by UOB and the successful preparation of UOHP/AgNP composite coatings after heat curing.

Afterwards, XPS analysis on the surface of UOHP/AgNP composite coatings was performed to provide further evidence for the presence of AgNPs on the composite coatings, as shown in Figure 3b,c. The XPS survey was first conducted to analyze the surface chemical composition of UOHP/AgNP composite coatings (Figure 3b), revealing two peaks with intensities at 287.3 eV and 538.0 eV, which assigned to C1s and O1s, respectively. Moreover, there is an additional signal peak with comparatively lower intensity at 404.0 eV, attributed to N1s. These results indicated that the surfaces of UOHP and UOHP/AgNP composite coatings contain a large amount of C and O elements, as well as a small amount of N elements. Due to the addition of Ag in small quantities, however, it is difficult to observe the peaks of Ag in the XPS survey, and only a weak Ag3d peak can be observed in the XPS survey of the UOHP-1.0%AgNP composite coating at 375.1 eV. Further, according to the peak fitting procedure of the high resolution XPS spectra to account for the electronic structure of silver species (Figure 3c). UOHP coatings did not receive any Ag, thus the Ag3d spectra of the UOHP surfaces did not monitor any Ag signals. However, double peaks were clearly observed on the Ag3d spectra of UOHP/AgNP composite coating surfaces at 368.3 eV and 374.3 eV. The double peaks were attributed to the two spin-orbit of the Ag3d orbitals, Ag3d_5/2_ and Ag3d_3/2_, respectively. The intensity of the peaks in the Ag3d spectra was found to increase gradually with the increase in AgNP content. According to the literature [44,50], after fitting the spectra of Ag3d doublets, the Ag3d region has well-separated spin-orbit components (Δ_BE_ of Ag3d_5/2_ and Ag3d_3/2_ is 6.0 eV) and the binding energy of Ag3d_5/2_ in the Ag3d orbital is 368.2 eV. These results suggest that the silver is in the form of a metallic monomer rather than other forms of silver material. These results provided additional evidence of the monomeric silver content in UOHP/AgNP composite coatings and indicated the successful in situ reduction of Ag^+^ to AgNPs by UOB monomers.

**Figure 4:**
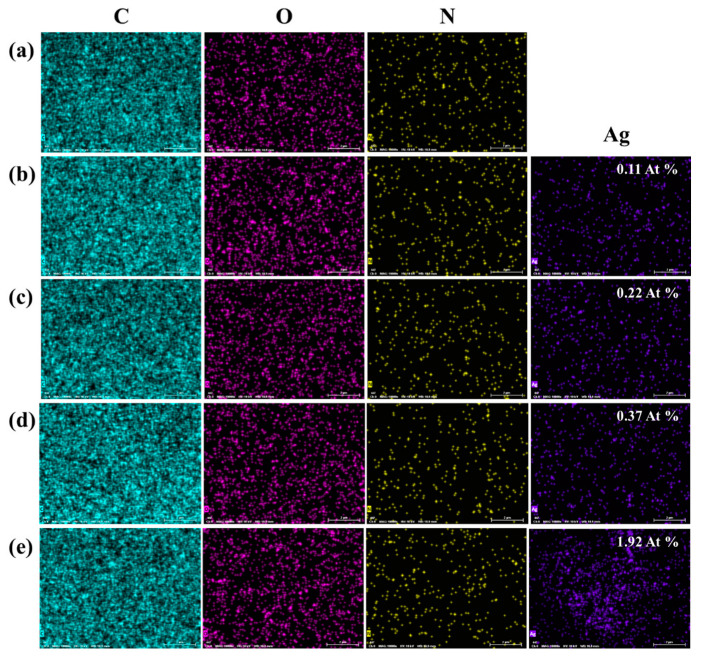
EDS analysis of surface chemical composition and mapping of (**a**) UOHP, (**b**) UOHP-0.05%AgNPs, (**c**) UOHP-0.1%AgNPs, (**d**) UOHP-0.5%AgNPs and (**e**) UOHP-1.0%AgNPs composite coatings. The information displayed at the bottom of each single image is as follows: MAG: 10,000×, HV: 10 kV, WD: 10.9 mm, and scale bar is 2 μm.

Meanwhile, EDS analysis was also performed to investigate the surface chemical composition of UOHP/AgNP composite coatings, as shown in Figure 4. The molecular composition of UOB monomer comprises mainly of four elements, C, O, N and H (Figure 1b). Consequently, the EDS elemental mapping of C, O and N elements suggest that all the elements are uniformly distributed in the measured region on the surface of the UOHP coating, which contains a large amount of C and O elements and a relatively lower amount of N elements. Obviously, the UOHP/AgNP composite coating also contains C, O and N elements, but with the addition of visible signals from Ag elements that are evenly dispersed on the composite coating’s surface. Furthermore, the elemental content analysis indicates a gradual increase in the Ag elemental content in UOHP/AgNP composite coatings of 0.11 At%, 0.22 At%, 0.37 At% and 1.92 At%, respectively. These results suggest that the composite coatings’ surfaces contain AgNPs, and the AgNPs content rises with the additional amount.

**Figure 5:**
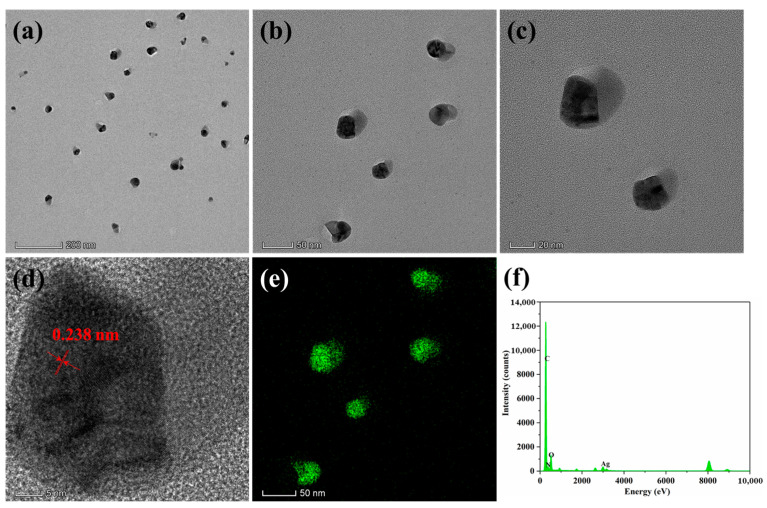
HR-TEM images of UOHP-1.0%Ag composite coating at different magnification scales (**a**–**d**), (**e**) element mapping of AgNPs, (**f**) EDS analysis of UOHP-1.0%Ag composite coating.

High-resolution transmission electron microscopy (HR-TEM) was utilized to investigate the particle size of AgNPs produced by the in situ reduction, as shown in Figure 5. The small-sized spherical-like shape of AgNPs was observed to be uniformly deposited on the UOHP-1.0%AgNP composite coating at different magnifications, with a particle size of approximately 20 nm. The crystal lattice fringe spacing of 0.238 nm was measured (Figure 5d), revealing the cubic crystal lattice structure with (111) plane of metallic silver [20,51]. The TEM elemental mapping analysis reveals that the Ag elements are uniformly distributed (Figure 5e). The EDS spectrum shows obvious Ag elements, indicating the presence of a silver monomer within the UOHP-1.0%AgNP composite coating (Figure 5f). These results further confirmed the in situ reduction of AgNPs by the UOB monomer, leading to the formation of UOHP/AgNP composite coatings through heating ring-opening polymerization.

### 3.3. Thermal Stability of UOHP/AgNP Composite Coatings

**Figure 6:**
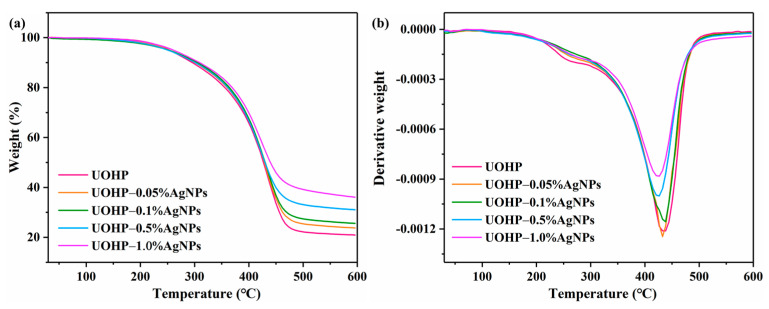
(**a**) TGA and (**b**) DTG curves of UOHP and UOHP/AgNP composite coatings.

TGA profiles were recorded to investigate the thermal stability of UOHP/AgNP composite coatings under nitrogen atmosphere, in terms of the initial decomposition temperature (T_5%_, which defined as the temperature for 5 wt% decomposition) and char yield. The results of TGA and DTG are shown in Figure 6, and the corresponding thermal stability parameters are listed in Table 2. Based on the experimental results, the initial decomposition temperature (T_5%_) and the maximum weight loss temperature (T_max_) of UOHP/AgNP composite coatings demonstrate no significant difference compared to that of the UOHP coating. The observation suggests that the preparation of AgNPs by the in situ reduction in the coating does not lead to notable variations in the thermal stability of the composite coatings, and apparently, the thermal stability of UOHP/AgNPs composite coatings remains similar to that of UOHP coating. But the experimental results show significant changes in the char yield of UOHP and UOHP/AgNP composite coatings. It is found that the residue of 20.9 wt% is left for UOHP at the end of the test, while the residue of 23.7 wt%, 25.6 wt%, 31.0 wt% and 36.1 wt% for UOHP-0.05%AgNP, UOHP-0.1%AgNP, UOHP-0.5%AgNP and UOHP-1.0%AgNP composite coatings, respectively. The char yield of UOHP/AgNP composite coatings gradually increases with the incorporation of AgNPs, therefore indicating a higher content of AgNPs in the composite coatings as the char yield improves.

### 3.4. Surface Properties of UOHP/AgNP Composite Coatings

**Figure 7:**
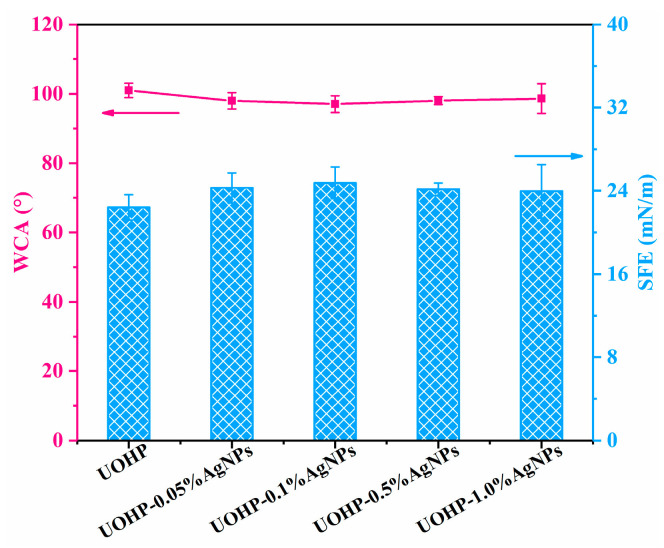
The WCA and SFE of UOHP and UOHP/AgNP composite coatings.

The WCA and SFE were measured in the atmospheric environment to study the hydrophobicity and surface energy of UOHP/AgNP composite coatings, and the results are shown in Figure 7. As can be seen in Figure 7, for the UOHP coating, the WCA was 101.5° ± 3.1°, and the SFE was 22.0 ± 1.22 mN/m. However, the WCA for UOHP/AgNP composite coatings slightly decreased to 98.0° ± 2.4°, 97.0° ± 2.4°, 98.0° ± 1.12° and 98.6° ± 4.3°, respectively. Meanwhile, the SFE exhibited an increasing trend, measuring at 24.3 ± 1.46 mN/m, 24.8 ± 1.53 mN/m, 24.1 ± 0.62 mN/m and 23.9 ± 2.60 mN/m. The alterations present on the surface of UOHP/AgNP composite coatings may account for these results. The AgNPs generated in situ can be located both deep within the coatings and on their surface. Consequently, the presence of AgNPs may change the initially flat and smooth UOHP coating surface and thus change the coating surface’s WCA and SFE. Nevertheless, UOHP/AgNP composite coatings remain as low-surface-energy surfaces, which synergize with the antimicrobial effect of Ag to effectively enhance the static antifouling performance for marine antifouling coating applications.

### 3.5. Fouling-Resistant Assays of UOHP/AgNP Composite Coatings

**Figure 8:**
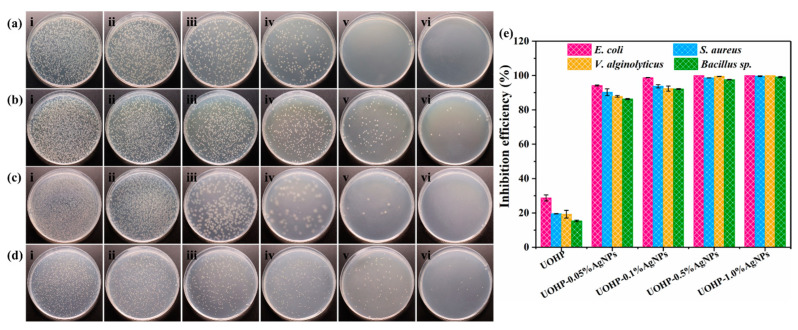
Digital photographs of antibacterial test towards typical (**a**) Gram-negative bacteria *E. coli*, (**b**) Gram-positive bacteria *S. aureus*, marine bacterial (**c**) *V. alginolyticus* and (**d**) *Bacillus* sp. after 24 h of incubation on (**i**) BG, (**ii**) UOHP, (**iii**) UOHP-0.05%AgNPs, (**iv**) UOHP-0.1%AgNPs, (**v**) UOHP-0.5%AgNPs and (**vi**) UOHP-1.0%AgNPs. (**e**) The inhibition efficiency of UOHP and UOHP/AgNP composite coatings relative to BG towards *E. coli* and *S. aureus*, *V. alginolyticus* and *Bacillus* sp.

AgNPs possess remarkable broad-spectrum antimicrobial properties, environmental friendliness, and negligible toxicity. The excellent antimicrobial properties originate from the minute size and the surface effects of AgNPs, and thus, a low dosage exhibits good antimicrobial properties, which can effectively inhibit and kill a wide range of bacteria. Therefore, the antimicrobial properties of UOHP/AgNP composite coatings were investigated using typical Gram-negative bacteria *E. coli* (BW25113), Gram-positive bacteria *S. aureus* (ATCC 25923) and the marine bacteria *V. alginolyticus* (ATCC 33787, Gram-negative bacteria) and *Bacillus* sp. (MCCC 1B00342, Gram-positive bacteria). The bactericidal ability was quantified by counting the number of bacterial colonies on agar plates and calculating the inhibition ratio of UOHP/AgNP composite coatings relative to the control sample made of the BG and UOHP coating, and results are shown in Figure 8. It is obvious that a large number of *E. coli*, *S. aureus*, *V. alginolyticus* and *Bacillus* sp. colonies can be seen on the agar plates cultured with bacterial isolates from BG. In addition, almost as many bacteria colonies were also isolated from the UOHP coating, and the inhibition ratios of the UOHP coating towards *E. coli*, *S. aureus*, *V. alginolyticus* and *Bacillus* sp. were 28.75 ± 1.77%, 19.5 ± 0.14%, 19.23 ± 2.24% and 15.35% ± 0.49%, respectively, in relation to the BG, indicating a UOHP coating without significant antibacterial properties. However, there were significant differences observed in the bacterial colonies present on the agar plates cultured with bacterial isolates from UOHP/AgNP composite coatings, with a notable reduction in the number of bacterial colonies isolated from the UOHP-0.05%AgNPs composite coating. The inhibition ratios of the UOHP-0.05%AgNP composite coating towards *E. coli*, *S. aureus*, *V. alginolyticus* and *Bacillus* sp. were 94.2 ± 0.28%, 90.28 ± 1.97%, 87.87 ± 0.52% and 86.23 ± 0.29%, respectively. Furthermore, the number of bacterial colonies on agar plates cultured with bacterial isolates from UOHP/AgNP composite coatings decreased as the amount of AgNPs increased. The UOHP-1.0%AgNP composite coating demonstrated the most significant antimicrobial activity towards *E. coli*, *S. aureus*, *V. alginolyticus* and *Bacillus* sp. under the current experimental conditions and the inhibition ratios were 100%, 99.65 ± 0.21%, 100% and 99.08 ± 0.31%, respectively, indicating that the UOHP-1.0%AgNP composite coating possesses superior antimicrobial properties.

Notably, the inhibition ratios of UOHP/AgNP composite coatings towards the four bacteria varied, and based on the above experimental results, it can be summarized that the UOHP/AgNP composite coatings generally showed stronger antimicrobial properties against Gram-negative bacteria than Gram-positive bacteria. The difference can be attributed to the dissimilarities in the physiological structures of the two major bacterial classes. The cell walls of Gram-positive bacteria have a dense and thick peptidoglycan layer composed of multiple layers and without lipids [52]. However, Gram-negative bacteria have a thinner and less cross-linked peptidoglycan layer compared to Gram-positive bacteria, along with a high amount of lipids [53]. It is widely accepted that the antimicrobial mechanism of AgNPs derive from the penetration of AgNPs and Ag^+^ through the bacterial cell membrane [54,55]. This intrusion disrupts the normal metabolism of bacteria, as well as the oxidative stress damage produced by AgNPs [54,56]. Gram-positive bacteria naturally have a thicker and denser cell wall, which hinders the penetration of AgNPs and Ag^+^ into the cell, hence providing stronger protection to the bacterial cell. Consequently, UOHP/AgNP composite coatings exhibit slightly lesser antibacterial efficacy against Gram-positive bacteria compared to Gram-negative bacteria.

**Figure 9:**
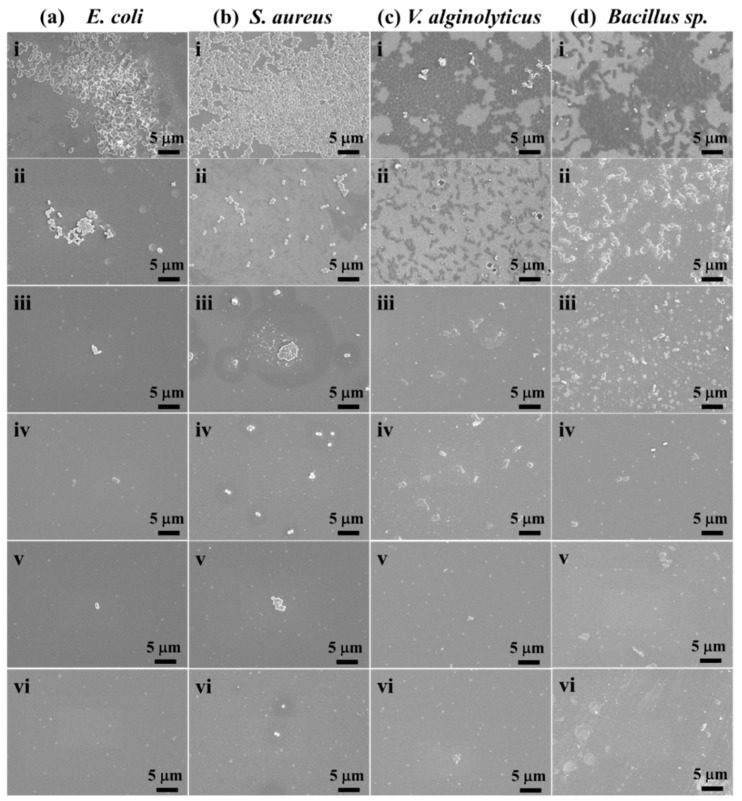
Adhesion of bacteria (**a**) *E. coli*, (**b**) *S. aureus* (**c**) marine bacterial *V. alginolyticus* and (**d**) *Bacillus* sp. after 24 h of incubation period on surface of (**i**) BG, (**ii**) UOHP, (**iii**) UOHP-0.05%AgNP, (**iv**) UOHP-0.1%AgNP, (**v**) UOHP-0.5%AgNP and (**vi**) UOHP-1.0%AgNP composite coatings by FE-SEM images (the scale bars are 5 μm).

In order to further determine the antibacterial performance of UOHP/AgNP composite coatings, the adhesion of bacteria on the surface of UOHP/AgNP composite coatings was visually examined by FE-SEM. The SEM images of *E. coli*, *S. aureus*, *V. alginolyticus* and *Bacillus* sp. adhered on different coatings are presented in Figure 9. A large number of tightly adhered *E. coli*, *S. aureus*, *V. alginolyticus* and *Bacillus* sp. cells can be observed on the surface of BG, while the bacterial adhesion on the surface of UOHP coating was significantly reduced and the UOHP/AgNP composite coating further reduced bacterial adhesion. It is obvious that the UOHP-0.5%AgNP and UOHP-1.0%AgNP composite coatings display excellent antimicrobial properties, with almost no adhesion of the four bacteria cells, which is consistent with the results of the antimicrobial rate. All four bacteria showed a large number of bacterial cells attaching to the surface of BG, indicating BG without antimicrobial activity. This is because BG lacks antimicrobial compounds, while it is made of materials possessing a high surface energy and modulus, which support bacterial settlement and adhesion, resulting in firm attachment to the surface that is difficult to rinse off with PBS. The UOHP coating has a low surface energy, resulting in weak bacterial adhesion, which can be easily washed away by PBS. Consequently, the adhesion of the four bacterial cells on the UOHP surface was significantly reduced. The UOHP/AgNP composite coatings with low surface energy also incorporate AgNPs which are highly effective in combatting bacteria by inhibiting the proliferation of bacteria cells and then reducing their quantity. The synergistic effect of AgNPs and the low-surface-energy property results in very few bacteria adhering to the surface of UOHP/AgNP composite coatings and decreasing gradually with the increase in AgNPs. Therefore, the settlement and adhesion of the four bacterial cells to the surface of UOHP-0.5%AgNP and UOHP-1.0%AgNP composite coatings was negligible.

**Figure 10:**
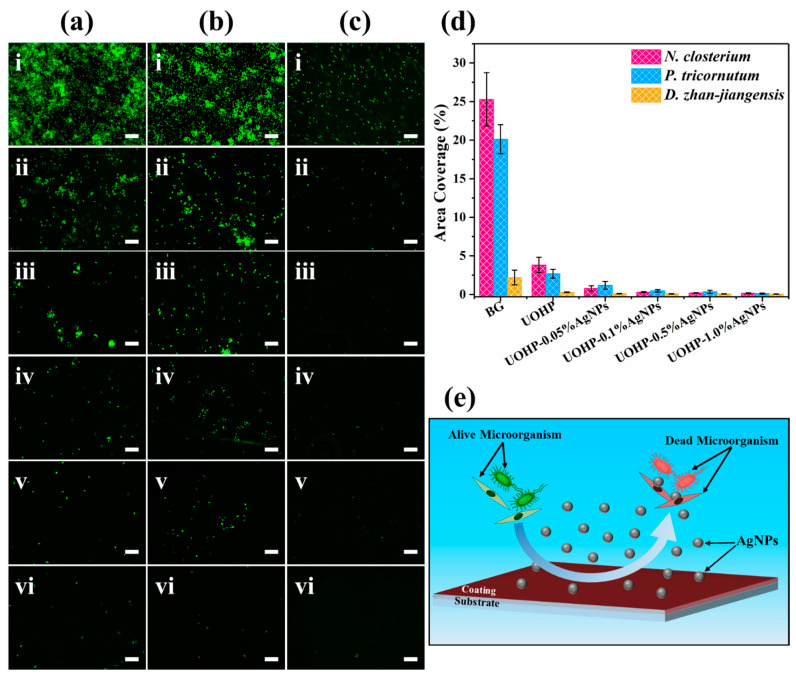
Fluorescent photographs of (**a**) *N. closterium*, (**b**) *P. tricornutum* and (**c**) *D. zhan-jiangensis* adhesion after 5 days of cultivation time on (**i**) BG, (**ii**) UOHP, (**iii**) UOHP-0.05%AgNPs, (**iv**) UOHP-0.1%AgNPs, (**v**) UOHP-0.5%AgNPs and (**vi**) UOHP-1.0%AgNPs composite coatings (the scale bars are 100 μm), (**d**) statistical chart showing algal density in examined fields by ImageJ 1.52a software, (**e**) antifouling mechanism of UOHP/AgNP composite coatings.

Marine microalgae are fouling organisms that settle on artificial surfaces in marine environments, forming conditioned biofilms that develop into biofouling. Therefore, microalgae such as *N. closterium*, *P. tricornutum* and *D. zhan-jiangensis* were used as model fouling species to investigate the anti-biofouling performance of the UOHP/AgNP composite coatings. Figure 10a–c display fluorescence microscopy images of microalgae adhered to the BG, UOHP and UOHP/AgNP composite coatings. Figure 10d shows the coverage area of microalgae on the coating surface after 5 days of immersion, analyzed by ImageJ 1.52a software. The uniform and brilliant fluorescence intensity indicates that a large number of microalgae had uniformly attached to the BG surface, which was highly conducive to microalgae attachment. The statistical analysis revealed that after immersing for 5 days, the coverage of *N. closterium*, *P. tricornutum* and *D. zhan-jiangensis* cells on the BG surface was 25.28 ± 3.46%, 20.12 ± 1.88% and 2.19 ± 0.96%, respectively. The fluorescence intensity on the surface of the UOHP coating was substantially weakened, signifying a reduction in the settlement and adhesion of marine microalgae cells. Compared to BG and UOHP, the fluorescence intensity of the surface of UOHP/AgNP composite coatings was reduced further, indicating a considerable decrease in the settlement and adhesion of marine microalga cells. In addition, the fluorescence intensity decreased as the AgNP content increased in the composite coatings, indicating a stronger inhibition of marine microalgae settlement and adhesion. Statistical analysis showed that the coverage of *N. closterium*, *P. tricornutum* and *D. zhan-jiangensis* cells on the surface of UOHP-1.0%AgNP composite coating after 5 days of immersion was 0.15 ± 0.06%, 0.11 ± 0.06% and 0.04 ± 0.02%, respectively, suggesting optimal inhibition of marine microalgae cells settlement and adhesion. The decrease in the settlement and adhesion of marine microalgae cells was primarily ascribed to the low-surface-energy characteristics of the coating, which facilitated the release of marine microalgae cells. Furthermore, the exceptional antimicrobial properties of the AgNPs within the coating and Ag^+^ released from the surface of AgNPs, which are toxic to microalgae cells [57,58,59], and thereby affecting the photosynthesis of microalgae cells, resulting in a reduction in their activity and proliferation, ultimately, demonstrating a greater anti-settlement and anti-adhesion capability [20]. It was also observed that the settlement and adhesion of *N. closterium* and *P. tricornutum* cells differed significantly from that of *D. zhan-jiangensis* cells on the surface. This distinction arises because *N. closterium* and *P. tricornutum* are benthic diatoms that naturally settle and accumulate on the coating surfaces. In contrast, *D. zhan-jiangensis* is not a benthic microalgae and instead uses its flagellum to swim in seawater, resulting in a decreased settlement and accumulation on the coating surfaces. Consequently, there is a significant difference in the amount of settlement and adhesion between the two benthic diatoms and the non-benthic microalgae.

## 4. Conclusions

A series of UOHP/AgNP composite coatings were fabricated by taking advantage of the phenolic hydroxyl groups present in the structure of urushiol-based benzoxazines, which generate free radicals with reducing properties, allowing for in situ reduction of AgNPs. The characterization results, including UV-Vis, XRD, XPS, EDS and TEM, demonstrate that AgNPs were successfully prepared by an in situ reduction in the coatings. Since AgNPs have broad-spectrum and highly effective antimicrobial properties, UOHP/AgNP composite coatings with a small amount of AgNPs (0.05 wt%) exhibit enhanced antimicrobial effects. Moreover, the antimicrobial effects are further enhanced with the increase in AgNP content. Notably, the antimicrobial effects of UOHP/AgNP composite coatings are significantly positively correlated with the increase in AgNP content. When the content of AgNPs reached 1 wt%, the UOHP/AgNP composite coatings demonstrated strong antimicrobial activity towards *E. coli*, *S. aureus*, *V. alginolyticus* and *Bacillus* sp. and the inhibition ratios were 100%, 99.65 ± 0.21%, 100% and 99.08 ± 0.31%, respectively. Similarly, the UOHP/AgNP composite coatings showed strong inhibition of the settlement and attachment of marine microalgae, further highlighting their outstanding antifouling performance. The exceptional antimicrobial performance of the UOHP/AgNP composite coatings was attributed to the synergistic effect of antifouling agent release and fouling release, which takes into account both static and dynamic antifouling performance. Given that AgNPs are both non-harmful to human and environmentally friendly, the UOHP/AgNP composite coatings can be regarded as a green and eco-friendly antifouling coating with long-lasting and high-efficiency antifouling performance and this work provides a technical support for the design of long-lasting, high-efficiency and eco-friendly antifouling coatings.

## Figures and Tables

**Table 1:** The specific compositions of UOB/AgNP composite coatings.

Samples	UOB (g)	AgNO_3_ (g)	Corresponding Polymer Nomenclature
UOB	10	0	UOHP
UOB-0.05%AgNPs	9.995	0.005	UOHP-0.05%AgNPs
UOB-0.1% AgNPs	9.99	0.01	UOHP-0.1% AgNPs
UOB-0.5% AgNPs	9.95	0.05	UOHP-0.5% AgNPs
UOB-1% AgNPs	9.90	0.1	UOHP-1% AgNPs

**Table 2:** Thermal-stability parameters of UOHP and UOHP/AgNP composite coatings derived from TGA and DTG curves.

Samples	T_5%_ (°C)	T_max_ (°C)	Residue at 600 °C (%)
UOHP	253.8	435.6	20.9
UOHP-0.05%AgNPs	253.7	432.8	23.7
UOHP-0.1% AgNPs	252.0	435.8	25.6
UOHP-0.5% AgNPs	250.8	436.2	31.0
UOHP-1% AgNPs	254.8	424.7	36.1

## Data Availability

The data presented in this study are available on request from the authors (due to privacy).
